# A population-level data linkage study to explore the association between health facility level factors and unintended pregnancy in Bangladesh

**DOI:** 10.1038/s41598-022-19559-w

**Published:** 2022-09-07

**Authors:** Md. Nuruzzaman Khan, Melissa L. Harris, Md. Nazmul Huda, Deborah Loxton

**Affiliations:** 1 Department of Population Science, Jatiya Kabi Kazi Nazrul Islam University, Mymensingh, Bangladesh; 2 Centre for Women’s Health Research, University of Newcastle, Callaghan, NSW Australia; 3 Translational Health Research Institute, School of Medicine, Western Sydney University, Campbeltown, NSW 2560 Australia

**Keywords:** Public health, Epidemiology

## Abstract

The objective of this study was to investigate the effects of health facility-level factors, including the availability of long-acting modern contraceptives (LAMC) at the nearest health facility and its distance from women’s homes, on the occurrence of unintended pregnancy that resulted in a live birth. We analysed the 2017/18 Bangladesh Demographic and Health Survey data linked with the 2017 Bangladesh Health Facility Survey. The weighted sample comprised 5051 women of reproductive age, who had at least one live birth within 3 years of the survey. The outcome variable was women’s intention to conceive at their most recent pregnancy that ended with a live birth. The major explanatory variables were the health facility level factors. A multi-level multinomial logistic regression model was used to assess the association of the outcome variable with explanatory variables adjusting for individual, household, and community-level factors. Nearly 21% of the total respondents reported that their most recent live birth was unintended at conception. Better health facility management systems and health facility infrastructure were found to be 14–30% protective of unintended pregnancy that resulted in a live birth. LAMC availability at the nearest health facility was associated with a 31% reduction (95% CI 0.50–0.92) in the likelihood of an unwanted pregnancy that resulted in a live birth. Health facility readiness to provide LAMC was also associated with a 14–16% reduction in unintended pregnancies that ended with a birth. The likelihood of unintended pregnancy that resulted in a live birth increased around 20–22% with the increased distance of the nearest health facility providing LAMC from the women’s homes.The availability of health facilities near women’s homes and access to LAMC can significantly reduce unintended pregnancy. Policies and programs to ensure access and affordability of LAMC across current health facilities and to increase the number of health facilities are recommended.

## Introduction

Unintended pregnancy is common worldwide, accounting for 48% of the world’s 250 million pregnancies^1^. Over 92% of unintended pregnancies—121 million in total—occur in low- and middle-income countries (LMICs)^1,2^, with an 11% increase in the last decade, from 109.4 million in 2000 to 121 million in 2019^2^. A significant percentage of unintended pregnancies end by abortion, mainly by unsafe abortion because of legal restrictions. It covers 4.7–13.2% of total maternal deaths in a year in LMICs^3,4^. Moreover, around 7 million admissions to the hospital in LMICs occur because of abortion-related complications (e.g., haemorrhage, sepsis, injury). This costs nearly 7 million USD in a year^3^. A similar trend is reported in Bangladesh, where around 47% of total pregnancies are unintended. However, after abortion, this figure represents around 25% of the total pregnancies that ended with live birth (over one million live births in a year)^4,5^. Approximately 647,000 unsafe abortions occur each year in Bangladesh, mainly outside the formal health facility (e.g. hospitals and clinics)^6,7^. Therefore, the subsequent medical consequences, including maternal mortality, are very high^8^. Unintended pregnancies are also associated with adverse birth outcomes, including stillbirth, miscarriage, and neonatal mortality^9–11^. These adverse outcomes are mediated mainly by the negative consequences of life events following unintended conception, such as depression and anxiety^9–11^, and the lower use of intrapartum, delivery, and post-partum care^12–16^. Consequently, unintended pregnancy is considered a serious public health threat in LMICs and Bangladesh^5,14,16^.

Although the unmet need for contraception is a critical factor in unintended pregnancy in LMICs^17–19^, over 30% of the total unintended pregnancies in LMICs occur due to the failure of contraceptive methods. The rate is slightly higher in Bangladesh, with over 33% of the total pregnancies^20^. There is also evidence that pregnancy due to contraceptive methods failure is increasing in Bangladesh^21^. This higher rate of contraception failure and related unintended pregnancy in LMICs and Bangladesh are mostly linked to policy-level challenges. For instance, the backlog effect of population momentum has created a “bulge” in the number of younger people in LMICs^5^. Although a significant improvement occurred because of successful implementation of the Millennium Development Goals (2000–2015) and ongoing target to ensure universal coverage of reproductive healthcare services by 2030 as part of the Sustainable Development Goals, the availability of contraception and the health facility preparedness to serve this growing population have not risen proportionally in LMICs^22^. Therefore, contraceptive uptake has been slowing in LMICs, and a higher number of contraceptive users are accessing less effective contraceptives, including condoms and pills (tier 2 and tier 2 contraception)^23^. The use of highly effective contraceptive methods and Long Acting Contraceptive Methods (LAMC, tier 1 contraception as classified by the WHO and the failure rate of this type of method is less than 1 per 100 women in 1 year, e.g. sterilisation, hormonal implants, intrauterine devices) are low in LMICs, including Bangladesh^24^. For instance, approximately 62% of the total women of reproductive age (15–49 years) in Bangladesh use contraception, while over 45% do not use highly effective contraceptive methods and over 40% use short-term modern contraceptives, including condoms and pills^4,8^. The failure rates of short-term methods in typical use are very high at 6–24%^25,26^. This burden exacerbates the existing 12% prevalence of unmet contraception needs—higher than the 10% prevalence in LMICs^4,8,27^.

Health facilities play a vital role in distributing LAMC and increasing its use in LMICs^4^. While the short-term modern contraceptives are available in the local market and distributed among women via family planning workers in LMICs and Bangladesh, the LAMC is only available in health facilities (e.g. government or private hospitals/clinics)^28,29^. Consequently, health facility-level factors, such as proximity, service quality, LAMC availability and health facility readiness to provide LAMC should be the crucial determinants of unintended pregnancy in LMICs and Bangladesh. However, the research conducted so far has focused on individual (e.g., women’s age, education, occupation), household (e.g., the number of children ever born, husband’s education and occupation and wealth quintile) and community (e.g., place of residence, place of the region)-level factors as contributing to unintended pregnancy^4,30–33^. Although there is evidence that the occurrence of unintended pregnancy is influenced by multiple factors (including health facility, individual-, households, and community-level factors) and the health facility-level factors plays a vital role there^5^, the role of health facility level factors on unintended pregnancy has not been examined so far. Therefore, this study aimed to determine the association between health facility-level factors and unintended pregnancy after adjusting for individual, household, and community-level factors.

## Methods

### Study overview

Data were extracted from the 2017/18 Bangladesh Demographic and Health Survey (BDHS) and linked with the 2017 Bangladesh Health Facility Survey (BHFS). The datasets were linked using geographical location variables collected during the surveys following the administrative boundary linkage method^34,35^. The National Institute of Population Research and Training conducted the surveys as part of the Demographic and Health Survey Program, with the assistance of the Ministry of Health and Family Welfare (MOHFW) of Bangladesh. Details relating to the sampling procedure of these surveys can be found elsewhere^28,29^.

The 2017/18 BDHS is a cross-sectional study and used multistage random sampling methods to collect a nationally representative sample. At the first stage of sampling, the survey selected 675 clusters. The Bangladesh Bureau of Statistics (an independent organization of the Bangladesh Government) canvassed 293,579 clusters (enumeration areas) as part of conducting the 2011 National Population Census (the most recent population census in Bangladesh). It includes one or more adjacent blocks, and the sum of all clusters covers all the areas of Bangladesh. Data were collected from 672 clusters, and the remaining three clusters were excluded due to flood. A household listing operation was conducted in each of the 672 clusters. This was then used at the second stage of sampling to select a fixed number of 30 households from each selected cluster through probability proportional to the unit size. A total of 20,160 households were selected, of which data collection was undertaken in 19,457 households with an over 96% inclusion rate. There were 20,376 eligible women in the selected households. The criteria used to select women were: (i) women aged 15–49 years of age who are usual residents of the selected household, or (ii) women who passed the most recent night before the day of the survey at the selected households. Of them, data were collected from 20,127 women with a response rate of 98.8%.

There were 19,811 registered health facilities in Bangladesh in 2017, according to the MOHFW. The 2017 BHFS used this list as a sampling frame and initially selected 1600 health facilities by systematic random sampling. A total of 1524 health facilities were finally included in the survey. For this, the survey included all available district health facilities (DHF, n = 62) and mother and child welfare centres (MCWC, n = 91). The reasons for including all the DHF and MCWC were fewer numbers of DHF and MCWC and the critical role they play in providing reproductive healthcare services, including family planning services and contraception. The remaining health facilities were selected by stratified random sampling of other government hospitals, private hospitals, and non-governmental organizations.

### Sample

We analysed data from 5051 women selected from 672 clusters included in the BDHS 2017/18. This number was extracted from the original sample of 20,127 women based on the following inclusion criteria: (a) had at least one live birth within 3 years preceding the survey and (b) reported their pregnancy intention at conception for this life birth.

### Outcome variable

Unintended pregnancy was measured by women’s intention to conceive at their most recent pregnancy that ended with live birth. Conception was categorized as wanted (planned or desired conception), mistimed (conception occurred earlier than expected but was wanted), or unwanted. We kept unwanted and mistimed pregnancies separate rather than consider them together because of the significant differences of their predictors. A similar classification has been used in previous research in Bangladesh^5,8,12–15^.

### Explanatory variables

The explanatory variables were selected based on a review of past literature^5,8,12–15^. The selected variables were broadly classified into four main groups in line with a multi-level analytic approach: health facility, individual, household, and community-level factors^36^.

The health facility-level variables were: (i) general health facility readiness (management and infrastructure), (ii) LAMC availability in the health facility, and (iii) and health facility preparedness to provide LAMC. We generated scores for the first two variables for the nearest health facility providing family planning and LAMC. The factors suggested by the World Health Organization (WHO) were used for this purpose, while the principal component analysis (PCA) was used to generate scores^37^. The third variable was generated by considering the general health facility reediness scores (management and infrastructure) and LAMC availability in the health facility score. Another health facility level variable considered was average regional level distance on-road communication from women’s usual residence to the nearest health facility. We used the average regional level distance rather than considering the actual distance because of health facilities included in the BHFS were sampled. Previous studies found in this approach women’s usual residence nearest health facility may not be included. Therefore, consideration of actual distance would be problematic^38,39^. The distance was calculated in two stages. First, the distances from each cluster to the health facility providing LAMC were calculated for each of the eight administrative divisions. This is then considered with the road communication system data of Bangladesh at the second stage and the average distance to women’s usual residence was computed per division. The details of these computation procedures can be found elsewhere^40,41^.

Individual-level factors included were women’s age at birth of the most recent child (≤ 19, 20–34, ≥ 35 years), women’s educational status (uneducated, primary, secondary, and higher), women’s working status (yes, no), parity (no children, 1–2, > 2), and preceding birth interval (≤ 2 years, 3–4 years, > 4 years).

Partner education (uneducated, primary, secondary, and higher), partner’s occupation (agricultural worker, services and non-agricultural labour, business, and others), family types (extended, nuclear), and wealth quintile (poorest, poorer, middle, richer, and richest) were included as household-level/family level factors.

The major community-level factors considered were women's place of residence (urban, rural), and division (Barisal, Chattogram, Dhaka, Mymensingh, Khulna, Rajshahi, Rangpur, Sylhet). Other community-level factors included were not directly available in the dataset used in the study. Therefore, these were constructed by aggregating the individual and household-level factors at the clusters’ level. They were community-level illiteracy and poverty, computed from the proportion of illiterate women and households living in the poorest and poorer wealth quintiles, respectively^42,43^.

### Statistical analysis

Descriptive statistics was used to describe the characteristics of the respondents. A multi-level multinomial logistic regression model was carried out to assess the associations of unintended pregnancy with health facility-level factors. The benefit of using a multi-level model is that it can analyse the clustering structure of BDHS’ data (individuals nested within a household and households nested within a cluster). Previous studies applied the multi-level modelling in similar data types and deemed this an appropriate approach^44,45^. The health facility level, individual, household and community-level factors mentioned in the explanatory variables section were included in the model by following the progressive model-building technique. Model 1 for individual-level factors. In Model 2, household-level/family level factors considered with the individual-level factors. Individual-, household-, and community-level factors were considered together in Model 3. Model 4 was the final model where each of individual, households, community, and health facility-level factors were included. We adjusted sampling weights for every analysis. Results were reported as relative risk ratios (RRR) with 95% confidence intervals (CI). All methods and analyses were performed according to the relevant guidelines. All analyses were performed using statistical package R (version 4.0.3).

### Ethical consideration

We analysed secondary data extracted from the Demography and Health Survey (DHS) program in de-identified form with permission to analyse. The survey questionnaire was reviewed by the National Research Ethics Committee of Bangladesh and ICF Macro International. They also provided the ethical approval. No other ethical approval is required to conduct this study. Informed consent was obtained from each respondent before interviewing.

## Results

### Socio-economic characteristics of the respondents

Table 1 shows the background characteristics of the respondents. Of the 5051 women included in the analyses, 1058 (20.88%) reported their most recent live births as being unintended at conception. The majority of women (70%) were aged between 20 and 34 years at the time of their most recent births. Around half of the women (49%) had secondary-level education. Around half of the total women had 1–2 child/children before the most recent index child. About 38% of women had not had any children. Over two-thirds of the women were residing in rural areas.

**Table 1:** Background characteristics of the respondents, Bangladesh, 2017/18.

	Overall distribution of the respondents (N = 5051)		Distribution of the respondents with unintended pregnancy (N = 1058)	
	Percentage	95% CI	Percentage	95% CI
**Individual level factors**				
Women’s age at birth of the last child				
≤ 19 years	25.07	23.71–26.48	19.02	16.55–21.76
20–34 years	70.72	69.26–72.15	72.56	69.46–75.46
≥ 35 years	4.21	3.65–4.84	8.42	6.78–10.41
Women’s education				
Illiterate	6.3	5.47–7.24	8.79	6.99–11.01
Primary	27.94	25.84–29.46	34.00	30.73–37.43
Secondary	48.99	47.17–50.82	42.99	39.62–46.43
Higher	17.19	15.63–18.67	14.22	12.00–16.77
Women’s working status				
Yes	37.31	35.16–39.50	42.51	38.78–46.33
No	62.69	60.50–64.84	57.49	53.67–61.22
**Household/family level factors**				
Partner’s education				
Illiterate	13.88	12.25–15.15	17.35	14.61–20.47
Primary	33.64	31.93–35.40	36.71	33.25–40.31
Secondary	33.99	32.35–35.68	30.82	27.55–34.31
Higher	18.49	16.98–20.93	14.96	12.65–17.56
Partner’s occupation				
Agriculture worker	19.13	17.52–20.82	22.50	19.59–25.71
Physical worker	52.34	50.43–54.22	51.24	47.64–54.83
Services	5.75	5.02–6.59	4.69	3.43–6.36
Business	20.36	18.96–21.89	21.12	18.33–24.20
Other	2.42	1.8–2.71	0.45	0.15–1.30
Parity				
1–2	80.22	78.55–81.79	66.65	62.97–70.14
> 2	19.78	18.21–21.45	33.35	29.86–37.03
Preceding birth interval				
≤ 2 years	6.75	5.98–7.60	15.72	13.37–18.41
3–4 years	17.85	16.67–19.10	30.82	27.80–34.01
> 4 years	75.40	73.96–76.79	53.46	50.04–56.85
Family types				
Nuclear family	31.35	29.68–33.07	24.67	21.62–28.00
Extended family	68.65	66.93–70.32	75.33	72.00–78.38
Wealth status				
Poorest	20.62	18.59–22.81	23.55	20.47–26.93
Poorer	20.51	19.0–22.09	24.31	21.48–27.37
Middle	19.19	17.67–20.81	16.75	14.25–19.59
Richer	20.16	18.42–22.01	19.61	16.82–22.73
Richest	19.53	17.62–21.59	15.78	12.82–19.29
**Community level factors**				
Place of residence				
Urban	26.85	15.17–28.59	28.57	25.21–32.19
Rural	73.15	71.41–74.83	71.43	67.81–74.79
Region of residence				
Barishal	5.70	5.14–6.33	6.75	5.59–8.14
Chattogram	21.20	19.54–22.96	16.64	13.92–19.78
Dhaka	25.60	23.88–27.40	25.64	21.94–29.73
Khulna	9.19	8.33–10.13	11.40	9.44–13.72
Mymensingh	8.53	7.68–9.47	7.99	6.51–9.77
Rajshahi	11.62	10.38–12.98	11.05	9.09–13.37
Rangpur	10.57	9.53–11.72	12.88	10.95–15.10
Sylhet	7.59	6.74–8.53	7.64	6.20–9.37
Community level illiteracy				
Low (≤ 20.0)	13.32	10.51–16.72	10.61	7.89–14.13
Medium (21.0–50)	65.35	60.77–69.66	68.86	63.47–73.78
High (> 50)	21.34	17.79–25.36	20.53	16.29–25.54
Community level poverty				
Higher (> 41.0%)	48.39	44.28–52.53	47.68	42.69–52.71
Medium (16.0–41.0)	24.52	20.75–28.71	26.16	21.70–31.18
Low (≤ 15.0)	12.84	10.12–16.17	12.05	9.20–15.64
Middle to richest community	14.25	12.14–16.66	14.11	10.96–17.98

Of the 1524 health facilities, 1357 (89%) provided LAMC (Table 2). The mean score for the variable general health facility readiness was 0.743 (0.620–0.850), and LAMC availability in the health facility was 0.892 (0.795–0.989). Around 82% (mean score: 0.818) (64.8–98.8%) of the total health facility found prepared to provide LMICs. The average distance between the health facilities and the BDHS clusters was 6.36 km, higher in the Sylhet division (8.34 km) and lower in the Dhaka division (4.83 km).

**Table 2:** Division-wise distribution of health facilities that provide long-acting modern contraception and their average distance from the demographic and health survey programme clusters, Bangladesh, 2017/18.

Division	Availability of long-acting modern contraception (N = 1524)		Average distance between home and health facility (km)
	Yes (n = 1357)	No (n = 167)	
Barishal	106 (93.71)	7 (6.29)	6.43
Chattogram	261 (90.49)	27 (9.51)	5.85
Dhaka	264 (87.07)	39 (12.93)	4.83
Khulna	175 (93.21)	13 (6.79)	5.92
Rajshahi	201 (91.54)	19 (8.46)	7.12
Rangpur	147 (76.36)	46 (23.64)	5.98
Sylhet	93 (96.13)	4 (3.87)	8.34
Mymensingh	110 (90.0)	12 (10.0)	6.44
Grand average distance			6.36

### Distribution of long-acting modern contraception use across women’s socio-economic characteristics

The distribution of unintended pregnancy across women’s socio-economic characteristics is presented in Table 1. A majority of the total unintended pregnancies were reported among women aged 20–34 years (73%), those who had a secondary level of education (43%), and those who had not engaged in formal income-generating activities (57.49%). Around 51% of the total unintended pregnancy was found to occur among women whose husbands were involved in physical work. We found almost 67% of the total unintended pregnancy among women with 1–2 children and those who had a birth interval of > 4 years (53%) between the second most recent live birth to the current index child.

### Associations between unintended pregnancy and health facility-level factors

The adjusted association of mistimed and unwanted pregnancy with health facility, individual, household and community-level factors (model 4 results) are presented in Table 3. In the supplementary file, we have presented the unadjusted effects of health facility-level factors on unintended pregnancy (supplementary Table 1) and results of all adjusted models (supplementary Table 2). The likelihood of a mistimed pregnancy was found to be reduced with the increase in scores of the health facility management system (RRR, 0.81, 95% CI 0.70–0.97) and health facility infrastructure (RRR, 0.88, 95% CI 0.78–0.99) (supplementary Table 1). The preparedness of the health facility that was closest to women’s homes to provide LAMC was found to have a protective effect on the occurrence of mistimed pregnancy (RRR, 0.87, 95% CI 0.72–0.99) and unwanted pregnancy (RRR, 0.91, 95% CI 0.81–0.99). We found an interdependence between the average distance of the nearest health facility from women's home and unintended pregnancy—for every kilometre (km) increase in distance, the occurrence of mistimed and unwanted pregnancy were found to be increased by around 23% (95% CI 1.01–1.47) and 28% (95% CI 1.03–1.61), respectively. These unadjusted relationships were most robust after adjusting different factors at the individual, household, and community levels (Table 3, supplementary Table 2). The variables, health facility management system (RRR, 0.84, 95% CI 0.70–0.96) and health facility infrastructure (RRR, 0.70, 95% CI 0.63–0.99), were found to be significantly associated with a reduced likelihood of unwanted pregnancy. However, these likelihoods were insignificant in the unadjusted model.

**Table 3:** Multi-level logistic regression modelling of unintended pregnancy and health facility level factors adjusting for individual, household/family, and community-level factors, Bangladesh, 2017/18 (N = 5051).

Factors	Mistimed pregnancy, RRR (95% CI)	Unwanted pregnancy, RRR (95% CI)
**General health service readiness**		
Health facility management system	0.79 (0.68–0.99)**	0.84 (0.70–0.96)**
Health facility infrastructure	0.86 (0.78–0.99)**	0.70 (0.63–0.99)**
**Long-acting modern contraception availability in health care facility**	0.86 (0.68–1.11)	0.69 (0.50–0.92)**
**Health care facility preparedness to provide long-acting modern contraception**	0.84 (0.71–0.96)**	0.86 (0.71–0.98)**
**Average distance to the nearest health facility provide long-acting modern contraception**	1.20 (1.03–1.46)**	1.22 (1.02–1.58)**
**Women’s age**		
≤ 19 (ref)	1.00	1.00
20–34	0.60 (0.47–0.78)***	1.86 (0.80–4.42)
≥ 35	0.40 (0.19–0.85)***	4.09 (1.58–6.58)***
**Women’ educational status**		
Primary (ref)	1.00	1.00
No education	1.45 (1.03–1.96)***	1.67 (1.07–1.96)***
Secondary	1.30 (0.79–2.16)	1.07 (0.67–1.75)
Higher	1.88 (1.07–3.32)	0.89 (0.44–1.81)
**Women’ working status**		
No (ref)	1.00	1.00
Yes	1.06 (0.87–1.29)	1.04 (0.80–1.36)
**Partner’s educational status**		
Primary (ref)	**1.00**	**1.00**
No education	2.19 (1.46–2.64)***	1.82 (1.58–2.15)***
Secondary	1.28 (0.91–1.80)	0.85 (0.58–1.24)
Higher	1.28 (0.91–1.80)	0.83 (0.46–1.50)
**Partner’s occupation status**		
Agricultural worker (ref)	1.00	1.00
Physical worker	1.10 (0.86–1.41)	0.78 (0.57–1.07)
Services	1.05 (0.64–1.73)	0.32 (0.13–0.80)***
Business	1.09 (0.81–1.45)	0.83 (0.57–1.20)
Other	3.02 (0.52–17.30)	3.08 (0.60–15.89)
**Number of children**		
No children (ref)	1.00	1.00
1–2	1.31 (0.97–1.77)	4.67 (3.13–6.69)***
> 2	1.02 (0.62–1.66)	5.69 (4.40–6.54)***
**Preceding birth interval**		
≤ 2 years	1.00	1.00
3–4 years	0.54 (0.39–0.75)***	0.68 (0.44–1.07)
> 4 years	0.12 (0.08–0.17)***	0.60 (0.39–0.90)***
**Family types**		
Nuclear (ref)	1.00	1.00
Extended	0.94 (0.77–1.15)	2.58 (1.79–3.71)***
**Wealth quintile**		
Poorest	1.11 (0.85–1.47)	1.05 (0.74–1.49)
Poorer	0.86 (0.63–1.19)	0.57 (0.37–0.87)***
Middle (ref)	1.00	1.00
Richer	0.86 (0.62–1.21)	0.74 (0.48–1.16)
Richest	0.67 (0.44–1.01)	0.45 (0.24–0.84)***
**Place of residence**		
Urban (ref)	1.00	1.00
Rural	0.83 (0.65–1.06)	0.92 (0.64–1.33)
**Division**		
Barishal (ref)	1.00	1.00
Chottogram	0.48 (0.32–0.70)***	0.40 (0.24–0.66)***
Dhaka	0.81 (0.54–1.21)	0.61 (0.36–1.04)
Khulna	1.31 (0.91–1.89)	0.85 (0.45–1.61)
Mymensingh	0.66 (0.45–0.98)***	0.68 (0.41–1.14)
Rajshahi	0.91 (0.62–1.34)	0.84 (0.46–1.52)
Rangpur	1.15 (0.80–1.64)	1.15 (0.70–1.87)
Sylhet	0.49 (0.33–0.72)**	0.48 (0.27–0.83)***
**Community-level illiteracy**		
Low (≤ 20.0)	1.00	1.00
Medium (21.0–50)	1.44 (1.03–2.00)***	1.02 (0.67–1.57)
High (> 50)	1.29 (0.86–1.93)	0.62 (0.36–1.06)
**Community-level poverty**		
Low (≤ 15.0) (ref)	1.00	1.00
Medium (16.0–41.0)	1.34 (1.05–1.71)***	1.64 (1.08–2.47)***
Higher (> 41.0%)	1.38 (0.95–2.00)	2.68 (1.58–4.54)***
Middle to richest community	1.26 (0.80–2.00)	0.32 (0.16–0.65)***
**Random effects**^**a**^		
Cluster-level variance (SE)^b^	0.03 (0.04)***	
Log-likelihood for fixed effects to random effects model	609.44***	
Log-likelihood ratio test for the null model to random effects model (chi-square)^c^	1624.59***	

^a^We assume that the within cluster-level random effects are equal for the ‘mistimed’ and ‘unwanted pregnancy’; therefore, only between cluster-level variance estimates are reported.

^b^Significance of random effects evaluated by comparing the model with a similar one in which random effects were constrained to zero.

^c^Compared to the null model with no-covariates. ***p < 0.01, **p < 0.05.

Of the different factors included in the model, women’s increasing age from 19 years or less was found to be protective of mistimed pregnancy (RRR, 0.40, 95% CI 0.19–0.85). This association was reversed for unwanted pregnancy, with 4.09 times (95% CI 1.58–6.58) higher risk of unwanted pregnancy among women aged 35 years and older than those aged 19 or younger. Women and their partners' educational status were found to be significant predictors of both mistimed and unwanted pregnancy. Compared to women with 1–2 children, the risk of unwanted pregnancy was 5.69 times (95% CI 4.40–6.54) higher among women with more than 2 children. Women with a greater than the 4-year interval between their most recent successive live births were less likely to have a mistimed (RRR, 0.12, 95% CI 0.08–0.17) or unwanted (RRR, 0.60, 95% CI 0.60, 95% CI 0.39–0.90) pregnancy.

Regarding household-level factors, women in the extended family were more likely to report unwanted pregnancies than those in the nuclear family. Women in the richest wealth quintile reported a 55% (95% CI 0.24–0.84) lower risk of unwanted pregnancy than those in the middle wealth quintile. Furthermore, increasing community-level illiteracy was found to be positively associated with the occurrence of mistimed pregnancy (RRR, 1.44, 95%, CI 1.03–2.00). Regarding community-level poverty, unwanted pregnancies tended to increase from around 1.64 (95% CI 1.08–2.47) times to 2.68 (95% CI 1.58–4.54) times with increased community-level poverty from low-poverty communities.

## Discussion

This study was conducted to determine the effects of health facility-level factors on the occurrence of unintended pregnancy. The closer the health facility was to the women’s residence, and the more prepared the facility was to provide LAMC, were found to be associated with 14–31% reduced likelihood of unintended pregnancy. A reciprocal increase in unintended pregnancy was found with increased distance between women's homes and the nearest health facility. These unique associations have not previously been demonstrated in Bangladesh and other LMICs and will inform policymakers about the need to improve health facility access to reduce the occurrence of unintended pregnancy.

We found that nearly 21% of the total live births in Bangladesh are unintended at conception, reduced insignificantly at around 5% from the 2014’s estimate^5,8^ and 10% from 2010’s estimate^46^. This reduction in the unintended pregnancy prevalence could be associated with the increase in LAMC use that occurred over the decade in Bangladesh, mainly among women who are advantaged in terms of wealth, education, and work engagement. The disadvantaged women (represent over 60% of the total country’s population) are still characterized with, (i) infrequent use of modern contraception^47^, (ii) ineffective contraception use^48^, (ii) inadequate use of emergency contraception^49^, or a combination of the three. Consequently, overall LAMC use remains the same in Bangladesh over the years^4,46,50^, and the occurrence of unintended is still very high in general and in disadvantaged groups in particular^20,30,31^. This situation is comparable to other LMICs^51–53^.

Being a major provider of modern contraception, mainly with a very minimal or free of cost, health facilities in Bangladesh and LMICs can play an important role in reducing the prevalence of unintended pregnancies, particularly among disadvantaged women. There are many reasons for this; however, financial burden and lower autonomy are important barriers^52,54,55^. For instance, the unavailability of modern contraception at the nearest health facility suggests that a couple might need to access contraception privately or collect contraceptives from a different health facility from a longer distance. Private purchase and travel are both barriers to use because couples (particularly disadvantaged couples) may not have the financial means to buy contraception. Also, couples may not be interested in accessing modern contraception from an unknown health facility, given that contraception is a culturally sensitive issue. In addition, women, who play a leading role in accessing and using contraception, may not have enough decision-making and financial autonomy to travel a longer distance to collect contraception^8,56^. These lead to a lower use of contraceptive methods, particularly LAMCs, which contribute to higher occurrence of unintended pregnancy. This observation is consistent with our study findings of higher likelihoods of unintended pregnancy with rising distance of nearest healthcare facility.

Given a constant lack of healthcare personnel, health facilities in Bangladesh are more engaged with providing curative care (e.g. treatment for delivery patients, pregnancy-related complications or other emergency care) than preventive care (e.g. family planning and contraception)^8,57^. Additionally, except for the specialized health facilities for family planning and maternal healthcare (such as Marie Stops Bangladesh), health facilities in Bangladesh mostly do not have private space to provide contraceptive counselling and LAMC^8,58^. Consequently, a significant proportion of the total healthcare facilities in Bangladesh are functionally ready to provide LAMC, however, in actual they do not provide LAMC^5^. Moreover, having MBBS and higher degrees, the healthcare providers at the healthcare facilities do not prefer to provide family planning and contraception services rather than providing curative care^8^. Besides these, mismatch between health facility management system and health facility infrastructure are common in Bangladesh. Consequently, available infrastructures do not properly used in many instances or health facility does not have enough infrastructures as per their requirement^5^. Furthermore, Bangladeshi health facilities have limited linkage with the family planning workers at the community level. However, they play a critical role in distributing short-term contraception and referring couples to a health facility for LAMC^5^. Therefore, health facilities are not well-informed about couple’s interests in accessing contraception and vice-versa^5,8^. These lead to a lower use of LAMC and, consequently a higher occurrence of unintended pregnancies.

Respondents’ individual, household, and community-level factors were also found to be important determinants of unintended pregnancy in Bangladesh—these findings are comparable with the studies conducted in others LMICs^8,59–61^. As reported in this study, these relationships can occur directly or indirectly through mediating the health facility-level factors^39^. We found that women aged 35 years or more and illiterate women were more likely to report unintended pregnancies. Similar findings were found in existing studies^5,20,30,31^. The major reasons are (a) older women may have already have their desired number of children, (b) they are less likely to use LAMC because of their lack of awareness and knowledge about the effectiveness of LAMC in preventing pregnancy, and (c) women’s lower autonomy to access health facilities^8,59^. Such barriers are even higher among women living with extended family and those residing in the community with higher poverty. Like other research on LMICs, we found a higher likelihood of unintended pregnancy among women living with extended family and residing in a community with higher poverty^8,62,63^. This study also found a regional level variation of unintended pregnancy occurrence: a higher likelihood of unintended pregnancy was found among couples residing in the Dhaka and Chattogram divisions. This finding confirms previous studies in Bangladesh^60,61^. This could be linked with the lower availability of the health facilities and their preparedness to provide LAMC in these divisions, findings reported in the previous studies of Bangladesh^4,64,65^. However, regional level variation of culture and economic development should not be overlooked as these also influence access to health facilities, LAMC use, and unintended pregnancy^5^. Some of the common forms of misconception are: (a) sons are preferable to daughters (as a son mostly look after parents when they are older, a son has greater earning capacity, and a son continues the patriarchal lineage) and, (b) use of contraception to prevent conception is equivalent to the killing of a human being^66^. Due to these misconceptions, many women in Bangladesh tend to follow religious norms and use limited contraceptives than expected^5^. Previous studies in Bangladesh substantiated that such misconceptions are higher among couples residing in the Sylhet and Chattogram divisions^5^ and among couples resided in thy communities increased community-level poverty^8^. These suggest the need for strengthening healthcare facilities and addressing these misconceptions through proper counselling by healthcare personnel and religious leaders, particularly among the disadvantaged group^39^. However, these are lacking in Bangladesh, particularly in the Sylhet and Chattogram divisions, where the number of contraceptive methods providing health facilities is lower than in other divisions of Bangladesh^39^. Similarly, there are currently no focus in the existing policy and program to prioritized the disadvantage community in proving contraceptive methods, particularly the LAMCs^5^.

There were several limitations to this study. This study’s data was extracted from a cross-sectional survey. Therefore, the findings presented in this study were correlational only, not casual. Besides, the BDHS displaced clusters’ locations up to 5 km for rural areas and 2 km for urban areas to ensure the privacy of the respondents. Therefore, the calculated average distance of the nearest health facility and its characteristics could be different from the actual distance. However, the BDHS ensured that displaced locations were placed within the same administrative boundary as the actual clusters’ locations. Therefore, such errors are minimal and likely to be random. Also, the data were based on participants’ self-reporting with no scope for validation by interviewers and may be subject to reporting errors. However, any such bias is likely to be random. Moreover, pregnancy intention data were collected retrospectively for births occurring 3 years prior to the survey, which might reflect recall bias and ambivalent responses in reporting pregnancy types. Furthermore, the data were collected only from women who had given birth, thus excluding data from women whose pregnancies were terminated. As specified, results pertain to women whose pregnancies were completed to birth. Regardless of these, this is the first study in LMICs and Bangladesh that determined influences of health facility-level factors on the occurrence of unintended pregnancy, adjusting with the individual, household, and community-level factors. The factors associated with the occurrence of unintended pregnancy were determined in multi-level framework adjusted with a wide range of factors. Therefore, the findings of this study are valid and could be applicable in making evidence-based policies and programs.

## Conclusion

Nearly 21% of all pregnancies in Bangladesh that end with live birth are unintended at conception, with significant variations across administrative divisions. Health facility-level factors were found as important predictors of unintended pregnancy in Bangladesh. Findings suggest the need for strengthening the existing health facilities to provide LAMC. Policies and programs are also recommended to ensure linkage among the community people, community family planning service providers, and health facilities. The provision of training for family planning service providers and the traditional providers at the community level and enlisting their help in distributing LAMC should also be considered outreach programs. Disadvantaged women need to be prioritized in every aspect of policies and programs.

## Supplementary Information

Supplementary Tables.

## Data Availability

The datasets used and analysed in this study are available from the Measure DHS website: https://dhsprogram.com/data/available-datasets.cfm.
