# Supplementary material for: A population-level data linkage study to explore the association between health facility level factors and unintended pregnancy in Bangladesh
Source: Sci Rep. 2022 Sep 7;12:15165. doi: 10.1038/s41598-022-19559-w (PMC9452522; doi:10.1038/s41598-022-19559-w)
Supplement: Supplementary file 1 — Supplementary Tables. [file 41598_2022_19559_MOESM1_ESM.docx]

**Supplementary table 1:** **Unadjusted** **multi-level multinomial logistic regression model with health facility level factors as the sole correlates of unintended pregnancy that ended with live birth (N=5,051)**

| **Health facility level factors** | **Mistimed pregnancy, RRR (95% CI)** | **Unwanted pregnancy, RRR (95% CI)** |
| --- | --- | --- |
|  |  |  |
| **General health facility readiness** |  |  |
| Health facility management system | **0.81 (0.70-0.97)^**^** | 0.90 (0.80-1.10) |
| Health facility infrastructure | **0.88 (0.78-0.99)^**^** | 0.86 (0.74-1.06) |
| Long-acting m**odern contraception availability in health care facility** | 0.86 (0.72-1.14) | **0.76 (0.60-0.80)^**^** |
| **Health care facility preparedness to provide long-acting modern contraception** | **0.87 (0.72-0.99)^**^** | **0.91 (0.81-0.99)^**^** |
| **Average distance to the nearest health facility provide long-acting modern contraception** | **1.23 (1.01-1.47)^**^** | **1.28 (1.03-1.61)^**^** |
| **Random effects^a^** |  | |
| Cluster-level variance (SE)^b^ | 1.51 (0.17)^***^ | |
| Log-likelihood for fixed effects to random effects model | 1114.94^***^ | |
| Log-likelihood ratio test for the null model to the random effects model (chi-square)^c^ | 74.06^***^ | |

**Notes:** ^a^We assume that the within cluster-level random effects are equal for the ‘mistimed’ and ‘unwanted pregnancy’; therefore, only between cluster-level variance estimates are reported.

^b^Significance of random effects evaluated by comparing the model with a similar one in which random effects were constrained to zero.

^c^Compared to the null model with no-covariates. ^***^*p<0.01, ^**^p<0.05*

**Supplementary table 2: Multi-level logistic regression modelling of unintended pregnancy and individual, household/family, community-, and health facility level factors, Bangladesh, 2017/18 (N=5,051)**

| **Factors** | Model 1  Individual level model | | Model 2  Individual and household level model | | Model 3  Individual, household, and community level model | | Model 4  Individual, household, community and health facility level model | |
| --- | --- | --- | --- | --- | --- | --- | --- | --- |
|  | Mistimed pregnancy, RRR (95% CI) | Unwanted pregnancy, RRR (95% CI) | Mistimed pregnancy, RRR (95% CI) | Unwanted pregnancy, RRR (95% CI) | Mistimed pregnancy, RRR (95% CI) | Unwanted pregnancy, RRR (95% CI) | Mistimed pregnancy, RRR (95% CI) | Unwanted pregnancy, RRR (95% CI) |
| **General health service readiness** |  |  |  |  |  |  |  |  |
| Health facility management system |  |  |  |  |  |  | **0.79 (0.68-0.99)^**^** | **0.84 (0.70-0.96)^**^** |
| Health facility infrastructure |  |  |  |  |  |  | **0.86 (0.78-0.99)^**^** | **0.70 (0.63-0.99)^**^** |
| Long-acting m**odern contraception availability in health care facility** |  |  |  |  |  |  | 0.86 (0.68-1.11) | **0.69 (0.50-0.92)^**^** |
| **Health care facility preparedness to provide long-acting modern contraception** |  |  |  |  |  |  | **0.84 (0.71-0.96)^**^** | **0.86 (0.71-0.98)^**^** |
| **Average distance to the nearest health facility provide long-acting modern contraception** |  |  |  |  |  |  | **1.20 (1.03-1.46)^**^** | **1.22 (1.02-1.58)^**^** |
| **Women’s age** |  |  |  |  |  |  |  |  |
| ≤19 (ref) | 1.00 | 1.00 | 1.00 | 1.00 | 1.00 | 1.00 | 1.00 | 1.00 |
| 20-34 | 0.88 (0.72-1.07) | **1.94 (1.65-2.24)^**^** | **0.58 (0.45-0.75)^**^** | 1.87 (0.81-4.33) | **0.60 (0.47-0.78)^***^** | 1.88 (0.80-4.41) | **0.60 (0.47-0.78)^***^** | 1.86 (0.80-4.42) |
| ≥35 | 0.42 (0.22-0.80) | **4.89 (3.63-6.13)^**^** | **0.39 (0.19-0.82)^**^** | **4.26 (1.68-6.84)^***^** | **0.39 (0.18-0.82)^**^** | **4.11 (1.59-7.12)^**^** | **0.40 (0.19-0.85)^***^** | **4.09 (1.58-6.58)^***^** |
| **Women’ educational status** |  |  |  |  |  |  |  |  |
| Primary (ref) | 1.00 | 1.00 | 1.00 | 1.00 | 1.00 | 1.00 | 1.00 | 1.00 |
| No education | 1.27 (0.79-2.02) | 0.82 (0.57-1.18) | 1.26 (0.78-2.04) | 1.14 (0.76-1.71) | 1.25 (0.78-2.01) | 1.15 (0.76-1.72) | 1.45 (1.03-1.96)^***^ | 1.67 (1.07-1.96)^***^ |
| Secondary | 1.23 (0.77-1.96) | **0.49 (0.33-0.70)^**^** | 1.35 (0.82-2.24) | 1.10 (0.69-1.76) | 1.30 (0.79-2.14) | 1.05 (0.65-1.69) | 1.30 (0.79-2.16) | 1.07 (0.67-1.75) |
| Higher | 1.37 (0.83-2.25) | **0.15 (0.08-0.25)^**^** | 1.87 (1.07-3.29) | 0.95 (0.48-1.91) | **1.82 (1.04-3.21)^**^** | 0.86 (0.43-1.73) | 1.88 (1.07-3.32) | 0.89 (0.44-1.81) |
| **Women’ working status** |  |  |  |  |  |  |  |  |
| No (ref) | 1.00 | 1.00 | 1.00 | 1.00 | 1.00 | 1.00 | 1.00 | 1.00 |
| Yes | 1.14 (0.95-1.36) | **1.31 (1.05-1.64)^***^** | 1.14 (0.94-1.39) | 1.08 (0.84-1.39) | 1.05 (0.87-1.28) | 1.05 (0.80-1.37) | 1.06 (0.87-1.29) | 1.04 (0.80-1.36) |
| **Partner’s educational status** |  |  |  |  |  |  |  |  |
| Primary (ref) |  |  | 1.00 | 1.00 | 1.00 | 1.00 | **1.00** | **1.00** |
| No education |  |  | 1.18 (0.86-1.63) | 0.86 (0.61-1.22) | 1.18 (0.86-1.62) | 0.83 (0.59-1.17) | **2.19 (1.46-2.64)^***^** | **1.82 (1.58-2.15)^***^** |
| Secondary |  |  | 1.28 (0.91-1.79) | 0.88 (0.61-1.28) | 1.29 (0.92-1.81) | 0.88 (0.60-1.27) | 1.28 (0.91-1.80) | 0.85 (0.58-1.24) |
| Higher |  |  | 1.37 (0.91-2.07) | 0.87 (0.48-1.57) | 1.34 (0.89-2.02) | 0.87 (0.48-1.57) | 1.28 (0.91-1.80) | 0.83 (0.46-1.50) |
| **Partner’s occupation status** |  |  |  |  |  |  |  |  |
| Agricultural worker (ref) |  |  | 1.00 | 1.00 | 1.00 | 1.00 | 1.00 | 1.00 |
| Physical worker |  |  | 1.09 (0.85-1.39) | 0.84 (0.62-1.14) | 1.10 (0.87-1.41) | 0.78 (0.57-1.06) | 1.10 (0.86-1.41) | 0.78 (0.57-1.07) |
| Services |  |  | 1.00 (0.61-1.65) | **0.38 (0.16-0.92)^***^** | 1.04 (0.63-1.71) | **0.33 (0.13-0.81)^**^** | 1.05 (0.64-1.73) | 0.32 (0.13-0.80)^***^ |
| Business |  |  | 1.04 (0.78-1.39) | 0.86 (0.60-1.24) | 1.09 (0.81-1.45) | 0.83 (0.57-1.20) | 1.09 (0.81-1.45) | 0.83 (0.57-1.20) |
| Other |  |  | 2.83 (0.74-4.92) | 1.92 (0.40-3.27) | 3.07 (0.54-4.32) | 3.02 (0.58-6.34) | 3.02 (0.52-17.30) | 3.08 (0.60-15.89) |
| **Number of children** |  |  |  |  |  |  |  |  |
| 1-2 |  |  | 1.00 | 1.00 | 1.00 | 1.00 | 1.00 | 1.00 |
| >2 |  |  | **1.36 (1.01-1.83)^**^** | **5.86 (3.84-7.88)^***^** | 1.32 (0.98-1.78) | **5.72 (4.32-6.73)^**^** | 1.31 (0.97-1.77) | **5.69 (4.40-6.54)^***^** |
| **Preceding birth interval** |  |  |  |  |  |  |  |  |
| ≤2 years |  |  | 1.00 | 1.00 | 1.00 | 1.00 | 1.00 | 1.00 |
| 3-4 years |  |  | **0.53 (0.39-0.73)^***^** | 0.70 (0.45-1.10) | **0.54 (0.40-0.74)^***^** | 0.69 (0.45-1.07) | **0.54 (0.39-0.75)^***^** | 0.68 (0.44-1.07) |
| >4 years |  |  | **0.13 (0.09-0.18)^***^** | 0.67 (0.44-1.02) | **0.12 (0.09-0.17)^***^** | **0.61 (0.40-0.92)^**^** | **0.12 (0.08-0.17)^***^** | **0.60 (0.39-0.90)^***^** |
| **Family types** |  |  |  |  |  |  |  |  |
| Nuclear (ref) |  |  | 1.00 | 1.00 | 1.00 | 1.00 | 1.00 | 1.00 |
| Extended |  |  | 0.92 (0.76-1.12) | **2.37 (1.65-3.39)^**^** | 0.96 (0.79-1.17) | **2.59 (1.80-3.72)^**^** | 0.94 (0.77-1.15) | **2.58 (1.79-3.71)^***^** |
| **Wealth quintile** |  |  |  |  |  |  |  |  |
| Poorest |  |  | 1.18 (0.52-1.47) | 1.10 (0.72-1.59) | 1.15 (0.85-1.32) | 1.08 (0.67-1.52) | 1.11 (0.85-1.47) | 1.05 (0.74-1.49) |
| Poorer |  |  | 0.89 (0.62-1.43) | 0.84 (0.42-1.15) | 0.68 (0.43-1.19) | **0.63 (0.39-0.93)^***^** | 0.86 (0.63-1.19) | **0.57 (0.37-0.87)^***^** |
| Middle (ref) |  |  | 1.00 | 1.00 | 1.00 | 1.00 | 1.00 | 1.00 |
| Richer |  |  | 0.98 (0.74-1.35) | 0.90 (0.86-1.88) | 0.87 (0.63-1.22) | 0.73 (0.47-1.14) | 0.86 (0.62-1.21) | 0.74 (0.48-1.16) |
| Richest |  |  | 0.77 (0.54-1.11) | 0.87 (0.53-1.44) | 0.68 (0.45-1.02) | 0.43 (0.23-0.80) | 0.67 (0.44-1.01^)^ | **0.45 (0.24-0.84)^***^** |
| **Place of residence** |  |  |  |  |  |  |  |  |
| Urban (ref) |  |  |  |  | 1.00 | 1.00 | 1.00 | 1.00 |
| Rural |  |  |  |  | 0.82 (0.64-1.04) | 0.92 (0.64-1.33) | 0.83 (0.65-1.06) | 0.92 (0.64-1.33) |
| **Division** |  |  |  |  |  |  |  |  |
| Barishal (ref) |  |  |  |  | 1.00 | 1.00 | 1.00 | 1.00 |
| Chattogram |  |  |  |  | **0.47 (0.32-0.68)^***^** | **0.40 (0.24-0.67)^**^** | **0.48 (0.32-0.70)^***^** | **0.40 (0.24-0.66)^***^** |
| Dhaka |  |  |  |  | 0.79 (0.54-1.18) | 0.61 (0.35-1.03) | 0.81 (0.54-1.21) | 0.61 (0.36-1.04) |
| Khulna |  |  |  |  | 1.29 (0.90-1.85) | 0.85 (0.45-1.61) | 1.31 (0.91-1.89) | 0.85 (0.45-1.61) |
| Mymensingh |  |  |  |  | **0.65 (0.44-0.95)^**^** | 0.68 (0.41-1.15) | **0.66 (0.45-0.98)^***^** | 0.68 (0.41-1.14) |
| Rajshahi |  |  |  |  | 0.88 (0.60-1.28) | 0.84 (0.46-1.51) | 0.91 (0.62-1.34) | 0.84 (0.46-1.52) |
| Rangpur |  |  |  |  | 1.11 (0.78-1.59) | 1.16 (0.71-1.89) | 1.15 (0.80-1.64) | 1.15 (0.70-1.87) |
| Sylhet |  |  |  |  | **0.48 (0.33-0.71)^***^** | **0.49 (0.28-0.84)^***^** | **0.49 (0.33-0.72)** | **0.48 (0.27-0.83)^***^** |
| **Community-level illiteracy** |  |  |  |  |  |  |  |  |
| Low (≤20.0) |  |  |  |  | 1.00 | 1.00 | 1.00 | 1.00 |
| Medium (21.0-50) |  |  |  |  | **1.52 (1.10-2.11)^**^** | **1.03 (0.67-1.58)** | **1.44 (1.03-2.00)^***^** | 1.02 (0.67-1.57) |
| High (>50) |  |  |  |  | 1.35 (0.91-2.01) | 0.64 (0.37-1.09) | 1.29 (0.86-1.93) | 0.62 (0.36-1.06) |
| **Community-level poverty** |  |  |  |  |  |  |  |  |
| Low (≤15.0) (ref) |  |  |  |  | 1.00 | 1.00 | 1.00 | 1.00 |
| Medium (16.0-41.0) |  |  |  |  | **1.31 (1.03-1.66)^**^** | **1.67 (1.11-2.51)^**^** | **1.34 (1.05-1.71)^***^** | **1.64 (1.08-2.47)^***^** |
| Higher (>41.0%) |  |  |  |  | 1.35 (0.95-1.93) | **2.71 (1.60-4.59)^**^** | 1.38 (0.95-2.00) | **2.68 (1.58-4.54)^***^** |
| Middle to richest community |  |  |  |  | 1.27 (0.81-1.99) | **3.23 (1.63-6.41)^**^** | 1.26 (0.80-2.00) | **0.32 (0.16-0.65)^***^** |
| **Random effects^a^** |  |  |  |  |  |  |  | |
| Cluster-level variance (SE)^b^ |  |  |  |  |  |  | 0.03 (0.04)^***^ | |
| Log-likelihood for fixed effects to random effects model |  |  |  |  |  |  | 609.44^***^ | |
| Log-likelihood ratio test for the null model to random effects model (chi-square)^c^ |  |  |  |  |  |  | 1624.59^***^ | |
